# Novel Picornavirus Associated with Avian Keratin Disorder in Alaskan Birds

**DOI:** 10.1128/mBio.00874-16

**Published:** 2016-07-26

**Authors:** Maxine Zylberberg, Caroline Van Hemert, John P. Dumbacher, Colleen M. Handel, Tarik Tihan, Joseph L. DeRisi

**Affiliations:** a Department of Biochemistry and Biophysics, University of California, San Francisco, San Francisco, California, USA; b California Academy of Sciences, San Francisco, California, USA; c U.S. Geological Survey, Alaska Science Center, Anchorage, Alaska, USA; d Department of Pathology, University of California, San Francisco, San Francisco, California, USA; e Howard Hughes Medical Institute, Chevy Chase, Maryland, USA

## Abstract

Avian keratin disorder (AKD), characterized by debilitating overgrowth of the avian beak, was first documented in black-capped chickadees (*Poecile atricapillus*) in Alaska. Subsequently, similar deformities have appeared in numerous species across continents. Despite the widespread distribution of this emerging pathology, the cause of AKD remains elusive. As a result, it is unknown whether suspected cases of AKD in the afflicted species are causally linked, and the impacts of this pathology at the population and community levels are difficult to evaluate. We applied unbiased, metagenomic next-generation sequencing to search for candidate pathogens in birds affected with AKD. We identified and sequenced the complete coding region of a novel picornavirus, which we are calling poecivirus. Subsequent screening of 19 AKD-affected black-capped chickadees and 9 control individuals for the presence of poecivirus revealed that 19/19 (100%) AKD-affected individuals were positive, while only 2/9 (22%) control individuals were infected with poecivirus. Two northwestern crows (*Corvus caurinus*) and two red-breasted nuthatches (*Sitta canadensis*) with AKD-consistent pathology also tested positive for poecivirus. We suggest that poecivirus is a candidate etiological agent of AKD.

## IMPORTANCE

Avian keratin disorder (AKD) is an increasingly common disease of wild birds. This disease, characterized by beak overgrowth, was first described in the late 1990s and has been spreading rapidly both geographically and in terms of host species affected. AKD decreases host fitness and can be fatal. However, the cause of the disease has remained elusive, and its impact on host populations is poorly understood. We found a novel and divergent picornavirus in 19/19 AKD-affected black-capped chickadees that we examined but in only 2/9 control cases. We also found this virus in 4 individuals of 2 other passerine species that exhibited symptoms consistent with AKD. Our data suggest that this novel picornavirus warrants further investigation as the causative agent of AKD.

## INTRODUCTION

**FIG 1:**
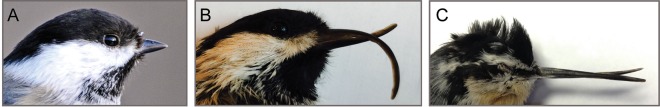
Avian keratin disorder. (A) An unaffected BCCH. (B) BCCH 971 (beak length: 18.7 mm). (C) RBNH 929 (beak length: 29.7 mm). (B and C) Both 971 and 929 exhibit beak overgrowth characteristic of AKD.

An epizootic of gross beak abnormalities was first documented among black-capped chickadees (BCCH [*Poecile atricapillus*]) in Alaska in the late 1990s (Fig. 1) (1). Birds suffering from this condition, termed avian keratin disorder (AKD), exhibit a variety of beak deformities (including elongation, crossing, and pronounced curvature) that interfere with the ability to feed and preen, behaviors vital for survival (1). In addition, affected birds exhibit changes in diet (2), changes in plumage structure and color (3), and higher rates of opportunistic infections (4) and avian malaria (5). As a result, affected individuals suffer fitness consequences, including decreased survival (1, 6). While data on the specific sources of morbidity and mortality are lacking, inability to feed and preen are presumed to lead to decreased fitness and mortality as a result of starvation, exposure to the elements, and infection with a variety of parasites and pathogens.

The population-level impacts of AKD remain unknown. However, infectious diseases can be an important selective force shaping the evolution, ecology, behavior, and community structure of host species (7). Infectious disease has also been recognized as an important factor in wildlife declines and extinctions (8). In particular, pathogens can (i) cause deterministic population declines directly if they kill hosts more quickly than hosts reproduce and (ii) suppress the size or growth rates of populations, making them more vulnerable to stochastic events. Small and otherwise sensitive populations are at particularly high risk from infectious diseases, and this threat increases dramatically when the pathogen infects multiple host species, which can then act as pathogen reservoirs (9). Infectious disease can also be problematic for species recovery programs (e.g., proventricular dilatation disease [PDD]), caused by a novel bornavirus, has hindered attempts to preserve the Spix’s macaw (*Cyanopsitta spixii*) (10).

The high prevalence, apparent high mortality rate, and widespread nature of AKD among multiple host species suggest that this pathology has the potential to have broad-ranging and negative impacts on wild bird populations. Epidemiological studies of AKD revealed that 6.5% of adult Alaskan BCCH are affected by AKD, a rate of beak deformities far greater than any previously known from wild birds, which typically exhibit frequencies of beak deformities of less than 1% (1). Meanwhile, similar deformities have been documented relatively frequently in numerous other species in Alaska and the Pacific Northwest region of North America, most notably among corvids; e.g., northwestern crows (NOCR [*Corvus caurinus*]), red-breasted nuthatches (RBNH [*Sitta canadensis*]), and raptors (1, 11; C. M. Anderson, personal communication to C. M. Handel). Morphologically similar deformities have been observed in more than 24 avian species in North America and 36 species in the United Kingdom (1, 12). However, despite the similarity of the gross pathology observed in multiple species, it remains unknown whether a common factor is responsible, in part because the cause of AKD remains elusive (4).

The identification of the causative agent of AKD will be key to determining whether the beak pathologies observed across species represent a multispecies pandemic. It is also a vital step toward evaluating the prevalence and impact of the disease, assessing population resistance, and developing an effective response to outbreaks, including the development of preventive policies, vaccines, or treatments as needed. There is a wide range of causes that can underlie beak deformities, including contaminants, nutritional deficiencies, trauma and infectious agents (13). Previous studies did not find clear evidence of a nutrient deficiency or contaminant exposure underlying AKD (4, 14, 15). Histopathological examination of the beaks of black-capped chickadee with AKD showed evidence of bacterial or fungal infection in a small portion of individuals examined, but the pattern of infection with these agents was not consistent across AKD-affected individuals (4). Transmission electron microscopy revealed features compatible with viral particles in some but not all affected birds (4). These observations were further supported by histopathological evidence of intranuclear inclusions in the beaks of AKD-affected but not unaffected birds, possibly indicative of a viral infection (4). These findings, along with the rapid emergence of this pathology both geographically and across species (1, 11) are consistent with an infectious etiology. However, the absence of a candidate agent has hindered progress in investigating this enigmatic disorder.

Metagenomic methods for the discovery of pathogens have revolutionized the ability to detect and identify candidate etiologic agents in the case of infectious diseases with unknown cause. We applied unbiased, high-throughput metagenomic sequencing to search for candidate causes of AKD. Using this methodology, we identified and assembled the complete coding region of a novel picornavirus, which we have named poecivirus after the genus of the host in which it was first detected. We then screened 19 AKD-affected BCCH and 9 control individuals for the presence of poecivirus by using targeted PCR primers followed by Sanger sequencing. In addition, we conducted opportunistic screening of two NOCR and two RBNH with AKD-consistent pathology for the presence of poecivirus. Here, we describe histopathological findings and tissue tropism, analyze the genome of this novel virus in the context of related viruses, and present data that identify poecivirus as a candidate etiological agent of AKD.

## RESULTS

### Metagenomic investigation of AKD.

**FIG 2:**
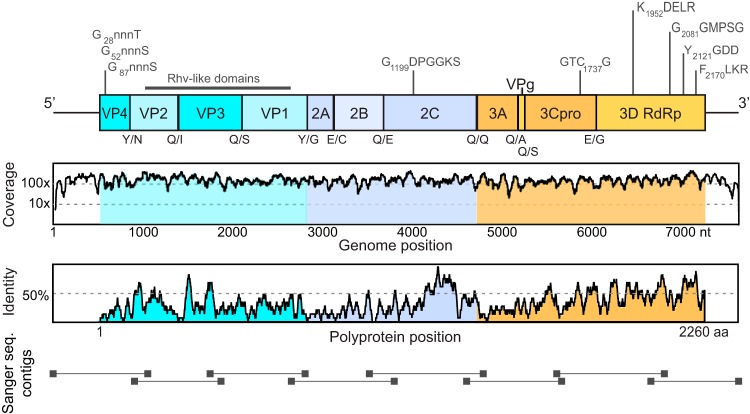
Poecivirus genome organization. (Top) Predicted genome organization. P1 (blue) represents viral structural proteins, and P2 (violet) and P3 (orange) represent nonstructural proteins. Predicted N-terminal cleavage sites are shown below the bar, and conserved picornaviral amino acid motifs are shown above it. (Middle) Number of reads from the metagenomic sequencing data set that support each base. (Bottom) Polyprotein homology between poecivirus and its closest relative, duck megrivirus, measured as the pairwise identity of a moving 15-amino-acid window.

Unbiased sequencing of pooled RNA from 8 individuals with AKD (Table 1) on the Illumina HiSeq platform generated approximately 1 × 10^7^ 50-nucleotide single-read sequences. We filtered the resulting reads (see Materials and Methods). We then assembled the remaining sequences into contiguous sequences (contigs) using the Trinity *de novo* assembler (16) and compared the assemblies to the NCBI nonredundant nucleotide (nt) and protein (nr) databases using BLAST. This search revealed 2 large contigs (3,299 nt and 4,560 nt) with similarities to avian picornaviruses (i.e., 21 to 27% pairwise nucleotide identity with pigeon mesiviruses). We manually assembled these contigs into a 7.6-kb sequence, accounting for most of the genome, including the full open reading frame (ORF), of a novel picornavirus (NCBI accession number KU977108). Retrospective mapping of the sequenced reads from the 8 AKD-positive beaks revealed that the pool of these samples contained approximately 21,000 viral sequences (0.04% of the total reads), with a mean viral-genome coverage of 139 reads. We validated the assembled sequence by Sanger sequencing (Fig. 2).

**TABLE 1:** Description and treatment of black-capped chickadees, red-breasted nuthatches, and Northwestern crows sampled

Species and specimen^a^	Yr	AKD status	Poecivirus present	Beak length (mm)	Beak appearance, histopathology			NGS^b^	Sample processing notes
					Elongated	Crossed	Normal, cellular-level hyperkeratosis		
BCCH									
160	2001	Affected	Yes	9.3	X			Yes	Full Sanger sequencing of ORF
263	2001	Affected	Yes	9.3	X			Yes	
394	2001	Affected	Yes	9.4	X			Yes	Full Sanger sequencing of ORF
449	2002	Affected	Yes	9.5	X			Yes	Full Sanger sequencing of ORF
451	2002	Affected	Yes	11	X			Yes	
478	2003	Affected	Yes	21.1	X	X		Yes	
498	2004	Affected	Yes	40.3	X	X		Yes	Full Sanger sequencing of ORF
600	2006	Affected	Yes	8.1	X			Yes	
738	2010	Affected	Yes	8			X		Full Sanger sequencing of ORF
953	2014	Affected	Yes	25.1	X	X			Stored at 4°C overnight prior to processing for poecivirus detection using virus-specific primers
954	2014	Affected	Yes	25.1	X				Stored at 4°C overnight prior to processing for poecivirus detection using virus-specific primers
955	2014	Affected	Yes	23.1	X	X			Poecivirus detection using virus-specific primers
956	2014	Affected	Yes	8.7	X				Stored at 4°C overnight prior to processing for poecivirus detection using virus-specific primers
967	2015	Affected	Yes	19.6	X	X			Stored at 4°C overnight prior to processing for poecivirus detection using virus-specific primers
968	2015	Affected	Yes	9.5	X	X			Stored at 4°C overnight prior to processing for poecivirus detection using virus-specific primers
970	2015	Affected	Yes	8.8	X				Stored at 4°C overnight prior to processing for poecivirus detection using virus-specific primers
971	2015	Affected	Yes	18.7	X	X			Stored at 4°C overnight prior to processing for poecivirus detection using virus-specific primers
972	2015	Affected	Yes	15.7	X	X			Poecivirus detection using virus-specific primers
973	2015	Affected	Yes	9.0	X	X			Poecivirus detection using virus-specific primers
183	1995	Unaffected	No	7.3					Poecivirus detection using virus-specific primers
389	2001	Unaffected	No	7					Poecivirus detection using virus-specific primers
392	2001	Unaffected	No	7.3					Poecivirus detection using virus-specific primers
401	2001	Unaffected	No	7.3					Poecivirus detection using virus-specific primers
460	2003	Unaffected	No	7.3					Poecivirus detection using virus-specific primers
473	2003	Unaffected	No	7.3					Poecivirus detection using virus-specific primers
596	2007	Unaffected	Yes	7					Full Sanger sequencing of ORF
601	2008	Unaffected	Yes	7.4					Full Sanger sequencing of ORF
739	2010	Unaffected	No	7.3					Poecivirus detection using virus-specific primers
RBNH									
12	1999	Affected	Yes	12.1	X	X			Poecivirus detection using virus-specific primers
929	2012	Affected	Yes	29.6	X	X			Poecivirus detection using virus-specific primers
NOCR									
674	2005	Affected	Yes	36.3	X				Poecivirus detection using virus-specific primers
848	2007	Affected	Yes	33.1	X	X			Poecivirus detection using virus-specific primers

a BCCH, black-capped chickadees; RBNH, red-breasted nuthatches; NOCR, northwestern crows.

b NGS, next-generation sequencing.

### Phylogenetic relationships.

**FIG 3:**
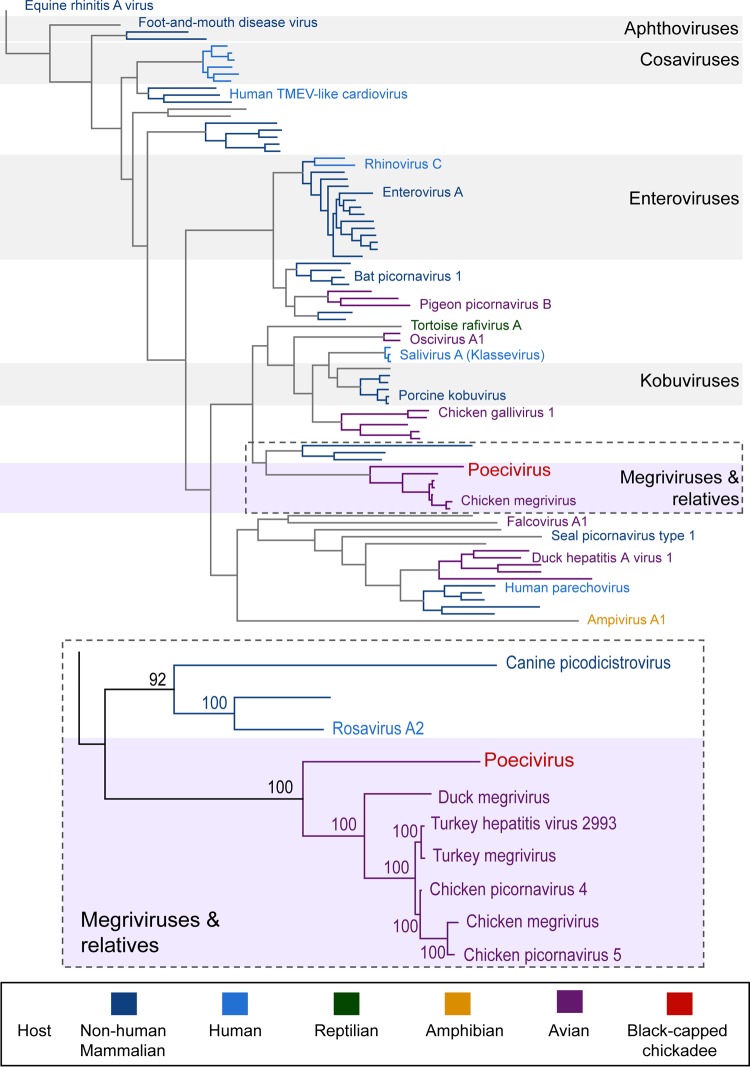
Phylogenetic tree of picornaviruses. (Top) Relationship between picornavirus polyproteins. Color indicates host taxa, with poecivirus highlighted in light red. TMEV, Theiler’s murine encephalomyelitis virus. (Bottom) Detail of boxed portion of top tree showing the relationship between poecivirus and its closest relatives; numbers indicate the percentage of bootstrap support for a given branch.

Phylogenetic analysis of the full polyprotein sequence (Fig. 3) and the 3C and 3D regions showed poecivirus to be a distinct species separate from classified genera and indicated that it is ancestral to the megriviruses, turkey hepatitis virus, and chicken picornavirus 4 and 5.

### Genomic organization.

Analysis of the recovered genome sequences revealed a nonsegmented positive-sense organization and a single ORF 6,783 nucleotides long (2,260 amino acids) (Fig. 2). Comparison of polyprotein sequences between BCCH hosts showed little interindividual variation, with viral sequences displaying 96.3% pairwise identity, indicating that the same viral species is present in all infected BCCH.

Poecivirus follows the typical picornavirus organization, with the single ORF coding for a polyprotein that includes the P1 region (structural proteins) and the P2 and P3 regions (nonstructural proteins involved in protein processing and genome replication) (17). To further investigate the relationship between this and previously described picornaviruses, we conducted comparative analyses of the ORF (Fig. 2). The predicted polyprotein is most closely related to those of avian picornaviruses, sharing 28.5%, 27.8%, and 27.7% amino acid identity with duck megrivirus and pigeon mesivirus 1 and 2, respectively. Possible cleavage sites of the polyprotein were Q/x and E/x (where lowercase x is a nonconserved amino acid) (Fig. 2); these cleavage sites are supported by NetPicoRNA (18) and by the alignment of the amino acid sequence to those of duck megrivirus and pigeon mesivirus 1 and 2 (NCBI accession numbers YP_009030047, AGS15016, and AGW95843).

### Analysis of the coding regions.

In the P1 region, the greatest sequence conservation between poecivirus and its closest relative, duck megrivirus, occurs in viral protein 3 (VP3) and VP0 (Fig. 2). Analysis of the P1 region did not support the presence of an L protein. This observation is in line with the reported lack of an L protein in pigeon mesiviruses and turkey hepatitis virus (NCBI accession number ADN94256) (19, 20). Alignments with sequences from close relatives (duck megrivirus and pigeon mesivirus 1 and 2) and NetPicoRNA (18) support cleavage of the P1 polyprotein at positions Y_112_/N, Q_293_/I, Q_531_/S, and Y_778_/G, resulting in the capsid proteins VP4, VP2, VP3, and VP1 (Fig. 2). The P1 region exhibited homology to the picornavirus capsid protein domain-like superfamily cluster cd00205 between amino acid residues 126 to 256, 382 to 518, and 558 to 717 (with E values of 4.02e−15, 2.57e−13, and 1.14e−06, respectively). VP0, the precursor of VP4 and VP2, contains multiple myristylation motifs typical of picornaviruses (Gxxx[T/S]), which are likely to be important for capsid assembly or entry of viral particles into host cells (21); these are located at G_28_, G_52_, and G_87_.

The P2 region of poecivirus is approximately 570 amino acids shorter than those of duck megrivirus and pigeon mesivirus, resulting in large part from differences in the 2A region. While duck megrivirus and pigeon mesivirus appear to have multiple 2A proteins (20, 22), poecivirus has a single 2A protein made up of just 97 amino acids, likely originating from a predicted cleavage from 2B at position E_875_/C. The poecivirus 2A protein does not exhibit sequence homology to any known picornavirus. The 2A protein does not contain the NPGP motif that facilitates C-terminal termination in aphthoviruses, avihepatoviruses, cardioviruses, erboviruses, senecaviruses, and teschoviruses (19). The poecivirus 2A protein also lacks the H box NC motifs seen in hepatoviruses, kobuviruses, parechoviruses, and tremoviruses (19). Neither amino acid sequence homology, structural modeling using HHPRED, nor manual inspection revealed a putative proteinase catalytic triad in the 2A protein. Therefore, the function of the 2A protein in this virus remains unknown. The 2B and 2C proteins are predicted to be processed by cleavage at the Q_1062_/E and Q_1408_/Q sites. The 2C region contains the highly conserved GxxGXGKS motif (where uppercase X represents an uncharged amino acid; G_1199_DPGVGKS) for NTP-binding sites but not the DDLxQ motif for putative helicase activity (23, 24).

Cleavage of the P3 region at sites Q_1559_/A, Q_1586_/S, and E_1852_/G is predicted to generate the 3A, 3B, 3C, and 3D proteins. Poecivirus 3A shows 23.2% and 23.8% pairwise identity to those of pigeon mesivirus 1 and 2 but no homology to that of duck megrivirus, its closest relative when considering the entire polyprotein sequence. The 3B protein contains a conserved tyrosine in position 3, which is present in the VPg proteins of all known picornaviruses and is required for viral RNA replication (17). The 3C protein contains the conserved motif GxCG (GTC_1737_G), which typically houses the active-site cysteine in picornaviruses (23) (Fig. 2). Comparison to similar sequences using structural modeling (HHPRED) suggests a putative catalytic triad of H_1625_ D_1644_ C_1737_. Not surprisingly, the 3D region, which forms the polymerase required for viral replication (17), exhibits the highest homology to those of other picornaviruses, with 50.2% pairwise amino acid identity to its closest relatives (pigeon mesivirus 1 and 2). The 3D protein sequence (amino acids 1863 to 2260) exhibits homology to the RNA-dependent RNA polymerases (RDRPs; family PF00680) and contains several highly conserved motifs present in RDRPs (KDELR, GGxPSG, YGDD, and FLKR; K_1952_DELR; G_2081_GMPSG, Y_2121_GDD, and F_2170_LKR) (Fig. 2).

### Prevalence, tropism, and histopathology.

Having characterized the sequence and genomic organization of poecivirus, we determined the infection status of 19 AKD-affected BCCH and 9 unaffected individuals using targeted PCR followed by Sanger sequencing (see Materials and Methods). Of the AKD-affected BCCH, 19 of 19 (100%) tested positive for poecivirus via PCR, while 2 of 9 (22%) unaffected individuals tested positive; thus, individuals with AKD were significantly more likely to be infected with poecivirus than expected by chance (*P* < 0.0001, likelihood-ratio chi-square test).

**FIG 4:**
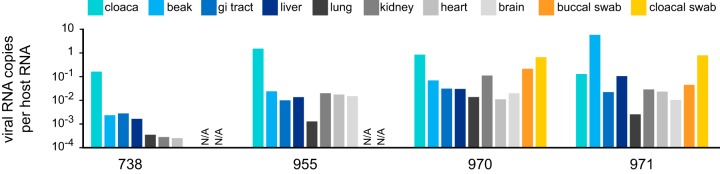
Viral tropism. Relative levels of viral RNA in different tissues from poecivirus-infected BCCH individuals were measured by qRT-PCR. Levels were normalized to levels of avian cellular RNA. Buccal and cloacal swabs were only tested for BCCH 970 and 971. gi, gastrointestinal.

When we tested multiple tissues to determine viral tropism, we detected poecivirus in all tissues sampled from each bird (Fig. 4). While it is not possible to draw conclusions regarding tropism given the small number of individuals sampled, it is noteworthy that the viral load was highest in either the cloaca or the beak, depending on the individual. Interestingly, cloacal swabs exhibited relative viral loads that were as high or higher than those in samples of cloaca tissue, suggesting that swabs provide a relatively noninvasive approach to sample individuals for infection with poecivirus. The viral loads in the two BCCH unaffected by AKD that tested positive for poecivirus were within the range of the viral RNA levels observed in AKD-affected BCCH; beak samples from individuals 596 and 601, both unaffected by AKD, contained 0.025 and 0.027 viral RNAs per host RNA (a level of viral RNA comparable to that of individual 955, affected by AKD).

**FIG 5:**
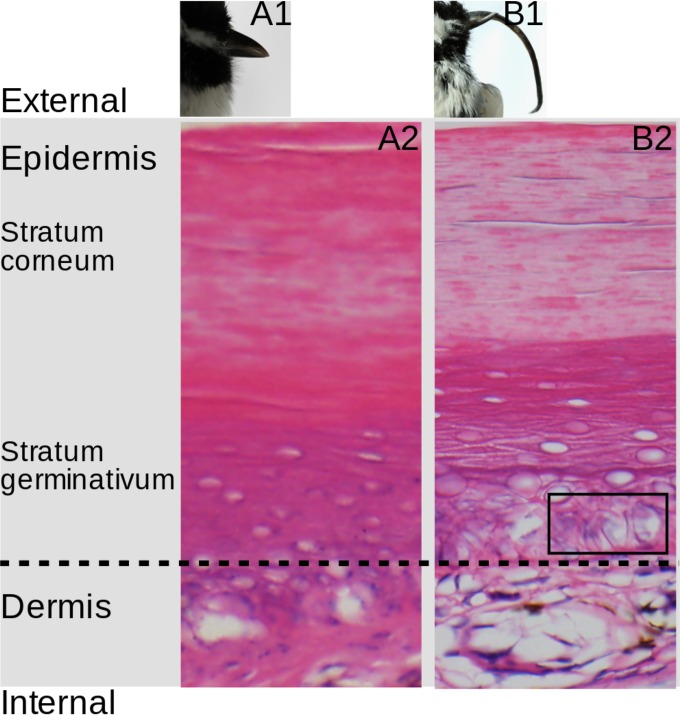
Histopathology of AKD. (A1 and B1) Gross beak morphology of BCCH 601 (unaffected by AKD) and 498 (AKD affected; beak length is 40.3 mm), respectively. (A2 and B2) Histopathology of the beaks of BCCH 601 (A2) and 498 (B2). The black box (B2) indicates an area of cytoplasmic vacuolization of cells; the nuclei of these cells show contour irregularities and areas surrounded by a rim of clear cytoplasm, creating an “owl eye” appearance.

As previously documented, histopathological examination of individuals with AKD showed hyperkeratosis, increased eosinophilia, and incomplete maturation of corneocytes (4). Individual 498, afflicted with the most extreme overgrowth of any BCCH in this study (with a beak of over 40 mm in length), exhibited koilocytotic keratinocytes, with nuclear irregularities and perinuclear halo (Fig. 5). Interestingly, vacuolated keratinocytes, or koilocytotic changes, are characteristic of virally induced cytopathic changes that accompany hyperkeratosis in verruca vulgaris (common warts caused by infection with human papillomavirus); however, we could not find evidence of papillomavirus in our metagenomic deep-sequencing data from AKD-affected BCCH.

### *In vitro* virus culture.

To further characterize this virus, we attempted to develop *in vitro* viral growth conditions using duck embryo, chicken embryo, and QT6 (quail) cells; however, we were unsuccessful. Viral RNA was initially detectable in the inoculum (prepared from virus-positive beak and other tissues) and supernatant but disappeared rapidly and remained undetectable for 72 days. This suggests that these cell lines or the culture conditions that we used were not permissive for viral replication, that the cell lines used might not express the receptor(s) used by poecivirus to gain entry into cells, or that the inoculum that we used did not contain replication-competent virus.

### Additional species.

The two NOCR and two RBNH with elongated beaks all tested positive for poecivirus via PCR using poecivirus-specific primers that target the 5′ untranslated region (UTR) and the P1 region. Sanger sequencing of the PCR products revealed virus with high nucleotide identity to the poecivirus detected in BCCH (NCBI accession numbers KU986726, KU986727, KU986728, KU986729, KU986730, KU986731, KU986732, and KU986733). The 5′ UTR regions displayed 92.4% (NOCR 674), 91.8% (NOCR 848), and 92.5% (RBNH 12 and 929) nucleotide identities with the BCCH poecivirus, while a 300-bp sequence in the P1 region displayed 92.0% (NOCR 674, 848), 91.0% (RBNH 12), and 91.3% (RBNH 929) nucleotide identities with the BCCH poecivirus. Across this region, virus from NOCR exhibited 24 mutations, of which 23/24 (95.8%) were synonymous with the virus detected in BCCH; meanwhile, the virus from RBNH exhibited 28 (RBNH 12) and 26 (RBNH 929) mutations, of which 27/28 (96.4%) and 25/26 (96.2%), respectively, were synonymous with the virus from BCCH. Furthermore, phylogenetic analysis of this region shows that the RBNH viruses were more closely related to each other than to viruses isolated from the other species and that the NOCR viruses were more closely related to each other than to viruses isolated from the other species. In addition, viruses isolated from RBNH and NOCR were more divergent from the BCCH-isolated poeciviruses than the BCCH viruses were from each other; this is consistent with the possibility of adaptation to particular host species.

## DISCUSSION

In this study, we used next-generation sequencing to investigate potential causes of avian keratin disorder (AKD), an emerging epizootic of beak deformities affecting numerous species across continents. Focusing on Alaskan BCCH, we found evidence of a single pathogen in the beaks of AKD-affected individuals: a novel picornavirus that we have named poecivirus. While we cannot rule out the presence of additional pathogens in other tissues, we did detect a strong, statistically significant correlation between poecivirus and AKD. Specifically, poecivirus was present in 19/19 (100%) AKD-affected BCCH and 2/9 (22%) control individuals. In addition to the correlation between poecivirus infection and AKD in BCCH, we detected poecivirus in two NOCR and two RBNH with elongated beaks. Importantly, poecivirus is not the first picornavirus associated with hyperkeratosis; Seneca Valley virus has been associated with hyperkeratosis in pigs (25).

Complete polyprotein analyses of poecivirus reveal a novel virus whose closest relatives are all avian picornaviruses, including megriviruses, turkey hepatitis virus, and chicken picornaviruses 4 and 5. While the polyprotein exhibits characteristic picornaviral organization and motifs, including picornavirus-like capsid domains and typical motifs in the 2C, 3C, and 3D functional domains, it is distinct from known picornaviruses. According to the Picornaviridae Study Group of the International Committee on Taxonomy of Viruses (http://www.picornastudygroup.com/definitions/genus_definition.htm), novel picornavirus genera are defined by amino acid identities in the P1, P2, and P3 regions of <40%, <40%, and <50%, respectively. Poecivirus exhibits ≤34%, ≤25%, and ≤48% identity to any known picornaviruses in the P1, P2, and P3 regions, respectively. This, along with lack of homology in the 2A, 2B, and 3A regions between poecivirus and its closest relatives indicates that poecivirus likely represents a new genus of picornavirus.

There are currently 26 genera in the *Picornaviridae* family, although knowledge of the family taxonomy is changing rapidly, with 9 new genera approved in 2014 alone. While the family includes 22 fully sequenced picornavirus species isolated from avian species, only a few these are known from wild bird species, including turdivirus 1 and 2 (26) and pigeon picornaviruses A and B (27). The known turdiviruses were found in samples from expired birds, and the pigeon picornaviruses were found during random sampling; as a result, we know little about the impacts of any picornavirus infection on wild birds. In domestic avian species, picornavirus infection can cause a variety of pathologies, ranging from subclinical infection to reduced growth rates to high mortality rates; however, it is primarily juvenile birds that are affected by these viruses (19, 28, 29). In contrast, AKD is documented primarily in adult birds, rarely causes overt disease in juveniles, and has not been documented in nestlings of either BCCH or other species studied (1, 11); however, symptoms other than beak overgrowth in juveniles and nestlings may have gone unnoticed. If poecivirus is the underlying cause of AKD, it is causing pathology in a manner substantially different from known avian picornaviruses.

Furthermore, if poecivirus is the cause of AKD across species, then it shows many of the hallmarks of a pathogen of concern from a conservation perspective. The available data suggest that mortality rates from this disease can be high among the adult breeding population; this could result in deterministic population declines if individuals are dying faster than they are replaced by recruitment of juveniles or migrants. In addition, avian picornaviruses characteristically decrease juvenile growth and survival (19, 28, 29); it remains unknown whether the causative agent of AKD has similar impacts on juveniles. If it does, this has the potential to limit recruitment of new individuals into breeding populations, making populations more vulnerable to stochastic events, such as high mortality due to adverse weather conditions. Finally, the apparently broad range of hosts affected by AKD suggests the potential for a large disease reservoir from which this pathogen could spill over into small, naive, or otherwise sensitive populations (9).

It is worth noting that two individuals with apparently normal beaks tested positive for poecivirus. This could indicate that poecivirus is not related to AKD; however, poecivirus was present in significantly more AKD-affected birds than unaffected individuals, suggesting that either it is the causative agent of AKD or AKD promotes infection with poecivirus. Certainly, definitive identification of individuals affected by AKD remains a challenge; the appearance of a gross beak deformity is the primary means of diagnosis, and yet, given the time required for a beak to become measurably elongated, the causative agent must be present before visible signs of deformity have appeared. As a result, it is possible that the two apparently unaffected birds that tested positive for poecivirus may have been in an early stage of infection, before sufficient time had passed for them to grow the elongated beak that defines AKD. Indeed, while 18 of the 19 individuals in this study that were classified as being affected by AKD had clearly deformed, elongated beaks, the remaining individual had a long but not obviously deformed beak and evidence of AKD at the cellular level (individual 738). All 19 of these individuals tested positive for the presence of poecivirus. It is also possible that some individuals exhibit subclinical infection (as has been suggested for related viruses [28]) or that coinfection with other pathogens or some other environmental trigger is needed to initiate the overgrowth of the beak keratin. For example, in the case of turkey viral hepatitis, thought to be caused by a picornavirus, clinical symptoms only become overt when individuals are stressed (19).

AKD is an emerging disease of wild birds that has been increasing both in geographic scope and in the variety of species affected. The strong association between poecivirus and AKD in BCCH suggests that this is a promising candidate for the causative agent of AKD. It is noteworthy that AKD appears to affect an incredibly wide range of avian species. Several lines of evidence (i.e., the concurrent emergence of AKD across species and the geographic proximity of such clusters) lend support to the idea that multiple species are being afflicted by a disease with a common etiology. In keeping with this, we found poecivirus to be present in 4/4 individuals with overgrown beaks from 2 additional species (NOCR and RBNH). However, control individuals from these species were not available for testing at the time of this study; screening of additional species with AKD-like deformities for the presence of poecivirus and sequencing of poecivirus from a greater variety of species will be necessary to further test the association between poecivirus and AKD. In addition, next-generation sequencing would be required to obtain the full viral genome from each of these hosts to confirm that they are infected by poecivirus, as opposed to a related but distinct species of virus. Finally, viral challenge experiments will be required to demonstrate with certainty that poecivirus is the cause of AKD. In the absence of an *in vitro* system for growing wild-type virus, one possible alternative would be to use *in vitro*-transcribed RNA derived from a full-length cDNA of the poecivirus genome to transfect cultured cells; this would bypass a potential block to viral entry of cultured cells if, for example, cells used in culture lacked the appropriate receptor.

In conclusion, the association between poecivirus and AKD warrants further investigation. The use of cloacal and/or buccal swabs to test individuals for infection with poecivirus provides a nonterminal method of sampling individuals for poecivirus infection that will make sampling large numbers of AKD-affected individuals substantially more feasible. The sampling of larger numbers of individuals, together with metagenomic characterization of other affected species, the development of an *in vitro* culture system, and viral challenge of healthy individuals will provide the information necessary to determine with certainty the clinical contribution of poecivirus to AKD and to elucidate both the pathogenesis and transmission potential of this virus. Ultimately, identifying the causative agent of AKD is vital in order to monitor the impact of this disease on wild bird populations, understand the causes underlying current disease emergence, and determine the possibility of and necessity for interventions.

## MATERIALS AND METHODS

### Study species.

The majority of the research documenting, describing, and investigating AKD has been conducted in BCCH (1, 2, 4, 6, 14). Furthermore, AKD has been documented in thousands of BCCH; this is an order of magnitude more affected individuals than have been documented in any other species (1). For these reasons, and because it is unknown whether similar deformities in other species represent the same pathology, we focus our investigation on BCCH.

### Sample collection.

Nineteen BCCH with AKD (Table 1) were trapped using funnel traps and mist nets in Anchorage and the Matanuska-Susitna Valley, Alaska, during the nonbreeding season from 2001 to 2015. Standard beak measurements were used to classify individuals as AKD affected or unaffected (1). Briefly, an individual was considered AKD affected if it had a nares-to-tip length (chord measurement from anterior end of the right nare to the tip of the upper beak) of ≥8.1 mm or evidence of hyperkeratosis at the cellular level. The 9 individuals with AKD that were collected from 2001 to 2010 were euthanized upon capture with isoflurane using the open-drop method and stored frozen at −20°C; the remaining 10 specimens were captured in the winter of 2014 and spring of 2015, euthanized, and stored overnight at 4°C prior to necropsy, at which time portions of tissues were frozen at −80°C and additional samples were placed in formalin. From 2 of these, we collected cloacal and buccal swabs prior to euthanasia (individuals 331 and 971); the swabs were stored in Longmire buffer (30). Nine individuals not affected by AKD were collected opportunistically between 1995 and 2010 and stored frozen at −20°C. To ensure that we did not classify individuals in the early stages of AKD as unaffected due to lack of gross morphological deformities, we examined beak tissue of individuals with apparently normal beaks via histopathology. As positive controls, we also examined the beaks of four AKD-affected individuals (individuals 600, 451, 263, and 498). Histopathological analysis was performed by T. Tihan and was done blind as to individual AKD status and picornavirus infection status. In addition, we opportunistically obtained two NOCR and two RBNH with overgrown beaks. One NOCR was submitted to a wildlife rehabilitation center in Alaska and euthanized, while the other crow was collected as part of airport safety measures. The two RBNH were collected after dying in the wild.

All work was conducted with the approval of the U.S. Geological Survey (USGS) Alaska Science Center Institutional Animal Care and Use Committee (assurance no. 2011-2) and under appropriate state and federal permits.

### Virus discovery: sample processing for high-throughput sequencing.

Tissue samples from 8 BCCH with AKD were processed for the initial metagenomic deep sequencing (Table 1). To extract RNA, half of a mandible from each individual was ground using a mortar and pestle on liquid nitrogen and then added to 1 ml of TRIzol reagent (Invitrogen) and processed following the manufacturer’s protocol. RNA quantity and quality were determined by spectroscopy. Comparable amounts of RNA from beak samples of each of the 8 BCCH were combined into a single pool and prepped for high-throughput sequencing using Illumina’s TruSeq RNA kit with the Ribo-Zero RNA kit.

### Sequence analysis.

Following default Illumina quality filtering, low-quality sequences containing any Ns and low-complexity sequences (Lempel-Ziv-Welch ratio of less than 0.45) were removed (31). The first 6 bases of each sequence were trimmed (these correspond to the random hexamer used to tag library molecules). Bird sequences were identified using the BLASTN alignment tool (version 2.2.21 [17]) to query a database composed of chicken, zebra finch, and quail genomes, and sequences aligning with an expected value of less than 10^−8^ were filtered. Similarly, sequences that aligned with the Illumina adapter sequences or to ϕX174 control sequence were removed. The remaining sequences were assembled into contiguous sequences using the Trinity *de novo* assembler (16). The resulting sequences were searched against the NCBI nonredundant nucleotide (nt) and protein (nr) databases. Only sequences whose best hits were to viral sequences were considered further. For coverage information, reads were aligned to Sanger-validated assemblies using the Bowtie2 software, version 2.0.0 (32). Analysis of the polyprotein for putative cleavage sites was conducted using the NetPicoRNA program, which predicts cleavage sites of picornaviral proteases (18), and by the alignment of the amino acid sequence to close relatives (i.e., duck megrivirus and pigeon mesivirus 1 and 2; NCBI accession numbers YP_009030047, AGS15016, and AGW95843). We also used the more sensitive HHPRED hidden Markov model-based alignment and structure prediction software tool to search for more distant homologies (33).

### Sanger sequencing.

The viral genome assembled from deep-sequencing reads was validated by Sanger sequencing (primers are listed in Table S1 in the supplemental material). Briefly, 200 ng of RNA was reverse transcribed in 10-µl reaction mixtures containing 100-pmol random hexamer, 1× reaction buffer, 5 mM dithiothreitol, 1.25 mM (each) deoxynucleoside triphosphates (dNTPs), and 100 U SuperScript III (Life Technologies); the mixtures were incubated at 25°C for 5 min, 42°C for 60 min, and 70°C for 15 min. The PCR mixtures contained 1× iProof master mix, 0.5 µM primer, and 5 µl cDNA. Thermocycling consisted of 98°C for 30 s, 40 to 45 cycles of 98°C for 10 s, 58°C for 10 s, and 72°C for 30 s, and a final elongation step of 72°C for 5 min. Amplicons were visualized using agarose gels; those in the correct size range were purified using a DNA Clean & Concentrator kit or Zymoclean gel DNA recovery kit (Zymo) and Sanger sequenced (Quintara Biosciences).

### Phylogenetic analysis.

All available picornavirus polyprotein and protein sequences were downloaded from NCBI. Multiple-sequence alignments were created using ClustalW alignment in Geneious (version 8.0.4) with free-end gaps; otherwise, default settings were used. Maximum-likelihood phylogenies were created using PhyML (PhyML plugin for Geneious version 2.2.0) with the LG model of amino acid substitutions, 100 bootstrap replicates, and otherwise default parameters (34).

### qPCR.

Quantitative PCR (qPCR) was used to determine viral tropism and monitor viral RNA levels in culture experiments. RNA was extracted with a Quick-RNA miniprep kit (tissue) or ZR viral RNA kit (tissue culture) (Zymo). RNA was reverse transcribed as described above; the qPCR mixtures contained 1× LC480 Sybr green master mix (Roche), 0.1 µM each primer (see Table S1 in the supplemental material), and 5 µl of 1:20-diluted cDNA.

### Tissue culture.

Duck embryo (CCL-141), chicken embryo (CRL-12203), and quail QT6 cells were obtained from the ATCC (American Type Culture Collection, Manassas, VA) and were grown at 37°C and 5% CO_2_. Duck embryo cells were grown in Eagle’s minimum essential medium (ATCC), while chicken embryo cells were grown in Dulbecco’s modified Eagle’s medium (ATCC); both were supplemented with 10% fetal bovine serum, 50 units/ml penicillin, and 50 µg/ml streptomycin. QT6 cells were grown in Ham’s F-12K medium (ATCC) with 2 mM l-glutamine adjusted to contain 1.5 g/liter sodium bicarbonate, supplemented with 10% tryptose phosphate broth, 5% fetal bovine serum, 50 units/ml penicillin, and 50 µg/ml streptomycin.

For inoculation experiments, portions of frozen, virus-positive beak, heart, brain, cloaca, kidney, liver, and ventriculus samples were minced with scalpels, suspended in serum-free DMEM containing 25 mM HEPES (pH 7.4), homogenized with a pellet pestle or Dounce homogenizer (beak), and placed on ice for 30 min. The homogenates were clarified by centrifugation at 10,000 × *g* for 1 min, filtered through a 0.2-µm filter, diluted 1:60, and used to inoculate cell cultures. The cells were visually monitored for evidence of infection, and the supernatants were monitored for the presence of viral particles using quantitative real-time PCR (qRT-PCR).

### Testing for an association between poecivirus and avian keratin disorder.

We tested for the presence of poecivirus in tissue samples from the 8 AKD-affected BCCH subjected to metagenomic deep sequencing, from 11 additional BCCH with AKD, and from 9 control BCCH. Half of a mandible from each black-capped chickadee was ground using a mortar and pestle on liquid nitrogen, and RNA was extracted using a Zymo Quick-RNA miniprep kit. In some cases, the cloaca was also tested; the cloaca was homogenized using a pellet pestle, and RNA was extracted using a Zymo Quick-RNA miniprep kit and reverse transcribed as described above. Samples were screened via PCR using poecivirus-specific primers (see Table S1 in the supplemental material), and amplicons were Sanger sequenced as described above, with a slight difference in the thermocycling: the thermocycling consisted of 98°C for 30 s, 40 to 45 cycles of 98°C for 10 s, 58°C for 10 s, and 72°C for 30 s, and then a 5-min elongation step of 72°C. Positive PCR results were confirmed by Sanger sequencing. We tested two NOCR and two RBNH with elongated beaks for the presence of poecivirus in the same manner, using primers BCCHpic_1F and BCCHpic_1R (see Table S1) and BCCHpic_2F and BCCHpic_2FR.

### Tropism.

Viral tropism was examined in four BCCH (individuals 738, 331, 955, and 971) via qPCR (as described above). We measured the levels of viral RNA relative to host RNA in the cloaca, beak, gastrointestinal tract, liver, lung, kidney, heart, and brain of all four individuals and in buccal and cloacal swabs collected from individuals 331 and 971. We also measured the levels of viral RNA relative to host RNA in the two BCCH that were unaffected by AKD but tested positive for poecivirus.

### Histopathology.

Tissues were fixed in 10% formalin, embedded in paraffin, cut into 5-µm sections, mounted on glass slides, and stained with hematoxylin and eosin.

### Nucleotide sequence accession numbers.

The sequence of poecivirus has been deposited in GenBank under accession number KU977108. The sequences obtained from the NOCR and RBNH have been deposited under accession numbers KU986726, KU986727, KU986728, KU986729, KU986730, KU986731, KU986732, and KU986733.

## SUPPLEMENTAL MATERIAL

Table S1  Poecivirus-specific primers. Primer BCCHpic_1F and BCCHpic_9R, targeting the 5′ UTR and 3′ UTR, respectively, are not represented in the Sanger sequencing-validated poecivirus genome deposited in GenBank (accession number KU977108). Table S1, DOCX file, 0.01 MB
