# Supplementary material for: Novel Picornavirus Associated with Avian Keratin Disorder in Alaskan Birds
Source: mBio. 2016 Jul 26;7(4):e00874-16. doi: 10.1128/mBio.00874-16 (PMC4958255; doi:10.1128/mBio.00874-16)
Supplement: Table S1 — Poecivirus-specific primers. Primer BCCHpic_1F and BCCHpic_9R, targeting the 5′ UTR and 3′ UTR, respectively, are not represented in the Sanger sequencing-validated poecivirus genome deposited in GenBank (accession number KU977108). [file mbo004162896st1.docx]

| **Primer name** | **Sequence** | **Target** |
| --- | --- | --- |
| BCCHpic_1F | AGCTTGGCCCCTCTAATTGT | 5’ UTR |
| BCCHpic_1R | GATTACTGTTCCGGTCTCTTGG | VP2 |
| BCCHpic_2F | CCAACTGAGTCGGATTCTACTG | VP2 |
| BCCHpic_2R | GTGAAACACCACCCAACATCA | VP3 |
| BCCHpic_3F | TGTCATACTTGCCACCTCCG | VP3 |
| BCCHpic_3R | CGACCGCATATCTTGTACGT | VP1 |
| BCCHpic_4F | TCGGACCCCAGTTAACTTGT | VP1 |
| BCCHpic_4R | TTTTACCGCACCAAACATGG | 2C |
| BCCHpic_5F | TGCTCCCACTGTTTCTCAAAG | 2B |
| BCCHpic_5R | CTTCGAGCACTTCAGTTGGG | 2B |
| BCCHpic_6F | GTTGTAGAGGCGGCAAAGAG | 2B |
| BCCHpic_6R | TACGAAAAGCCTCAGTCGGA | 3A |
| BCCHpic_7F | TGGCTGCTCTAGAGGATAAAGG | 2C |
| BCCHpic_7R | ACTGCACTACAACCAAATCTGT | 3C |
| BCCHpic_8F | CTTTGGGCTGCATGACAATT | 3C |
| BCCHpic_8R | CGGGTCCAAAACACATCTGG | 3D |
| BCCHpic_9F | GCTGGAAAGACTCGCGTAAT | 3D |
| BCCHpic_9R | TTGCTCCCCACCCATTGTAT | 3’ UTR |

Supplemental Table 1: poecivirus-specific primers. Primer BCCHpic_1F and BCCHpic_9R, targeting the 5’ UTR and 3’ UTR, respectively, are not represented in the Sanger sequencing validated poecivirus genome deposited in genbank (accession number KU977108).
